# Fear of Negative Evaluation Biases Social Evaluation Inference: Evidence from a Probabilistic Learning Task

**DOI:** 10.1371/journal.pone.0119456

**Published:** 2015-04-08

**Authors:** Katherine S. Button, Daphne Kounali, Lexine Stapinski, Ronald M. Rapee, Glyn Lewis, Marcus R. Munafò

**Affiliations:** 1 School of Social and Community Medicine, University of Bristol, Bristol, United Kingdom; 2 National Drug & Alcohol Research Centre, University of New South Wales, Sydney, Australia; 3 Centre for Emotional Health, Macquarie University, Sydney, Australia; 4 Mental Health Sciences Unit, University College London, London, United Kingdom; 5 Medical Research Council Integrative Epidemiology Unit at the University of Bristol, Bristol, United Kingdom; 6 UK Centre for Tobacco and Alcohol Studies, School of Experimental Psychology, University of Bristol, Bristol, United Kingdom; University of Granada, SPAIN

## Abstract

**Background:**

Fear of negative evaluation (FNE) defines social anxiety yet the process of inferring social evaluation, and its potential role in maintaining social anxiety, is poorly understood. We developed an instrumental learning task to model social evaluation learning, predicting that FNE would specifically bias learning about the self but not others.

**Methods:**

During six test blocks (3 self-referential, 3 other-referential), participants (n = 100) met six personas and selected a word from a positive/negative pair to finish their social evaluation sentences “I think [you are / George is]…”. Feedback contingencies corresponded to 3 rules, liked, neutral and disliked, with P[positive word correct] = 0.8, 0.5 and 0.2, respectively.

**Results:**

As FNE increased participants selected fewer positive words (β = −0.4, 95% CI −0.7, −0.2, *p* = 0.001), which was strongest in the self-referential condition (FNE × condition 0.28, 95% CI 0.01, 0.54, *p* = 0.04), and the neutral and dislike rules (FNE × condition × rule, *p* = 0.07). At low FNE the proportion of positive words selected for self-neutral and self-disliked greatly exceeded the feedback contingency, indicating poor learning, which improved as FNE increased.

**Conclusions:**

FNE is associated with differences in processing social-evaluative information specifically about the self. At low FNE this manifests as insensitivity to learning negative self-referential evaluation. High FNE individuals are equally sensitive to learning positive or negative evaluation, which although objectively more accurate, may have detrimental effects on mental health.

## Introduction

Fear of negative evaluation defines social anxiety [1] yet the cognitive processes involved in inferring social evaluation are poorly understood [2,3]. Cognitive biases are thought to maintain many psychiatric disorders, and while several areas of cognitive processing have been extensively studied in social anxiety, few studies have examined the role of instrumental learning. Social interactions are dynamic, with social behaviours contingent on evaluative feedback, which in turn is inferred from ambiguous social cues. Understanding how individuals use accruing cues to infer how others evaluate them may help to clarify the psychological mechanisms that underlie social anxiety [4].

Research into simple associative processes suggests social anxiety is associated with a loss of the positive associative bias observed in non-anxious controls [5–9]. However, none of these studies has examined instrumental learning as it might occur during a social interaction where the individual is inferring how the other social agent evaluates them using feedback. In a first attempt to address this, we found that individuals low in social anxiety favoured inferring positive evaluation relative to negative evaluation, making substantially fewer errors learning ‘the computer likes me’ than ‘the computer dislikes me’. This positive bias diminished as social anxiety increased driven by a decreased tendency to select words indicative of positive evaluation, and an increased tendency to select words indicating negative evaluation [4]. However, in our previous study we were unable to determine whether this effect was general to all social evaluation learning or was specific to social evaluation learning about the self. This issue has important clinical implications; treating a general bias requires a different approach to treating a highly selective one.

According to dual-process models, automatic processes, such as, for example, implicit associative learning which might occur in-situ during a social interaction, are functionally distinct from the global reflective processes which might occur before or after a social interaction [10]. Both are implicated in maintaining social anxiety [11–13]. Socially anxious individuals tend to conduct “post-mortems” after their social interactions, leading to a cycle of negative rumination [14,15]. In contrast, low anxious individuals tend to recall their past interactions with increasing positivity [14]. Understanding how FNE might be related to learning social evaluation in-the-moment during a social interaction may be important for understanding subsequent global reflections.

The present study extends our previous work by examining whether the social evaluation learning biases associated with FNE are specific to self-referential learning, and how they relate to global interpretations. To this end we developed a novel probabilistic social evaluation learning task which required participants to learn three social rules (person is liked, person is neither liked nor disliked, person is disliked) in both a self- and other-referential condition. Based on the research outlined above we tested the following hypothesis: FNE is associated with decreased endorsement of positive [and thus increased endorsement of negative] social-evaluative information, which is specific to learning about the self and not others.

## Method

### Participants

Participants were recruited via email from a database of research volunteers. Interested individuals completed a screening questionnaire including the Brief Fear of Negative Evaluation (BFNE) scale [16], and were eligible if aged 18–50 years, not currently receiving psychiatric medication, and spoke English as a first language or near-equivalent standard. One-third of participants were selected with BFNE scores in the lowest quartile (BFNE < 32), one-third from the highest (BFNE > 45) and the rest at random from the mid-range. In total, 100 participants provided data for analysis (low BFNE, *n* = 33; Mid BFNE, *n* = 32; high BFNE, *n* = 35).

### Ethics Statement

The study was conducted according to the Declaration of Helsinki (2008) and Good Clinical Practice guidelines, and was approved by the Faculty of Science Research Ethics Committee at the University of Bristol. Written consent was obtained from all participants. Participants were recruited from February to June 2011, and did not consent to their data being made open. The data that form the basis of the results presented here are available on request from the corresponding author.

### Materials

The social evaluation learning task used 64 word pairs comprised of semantically linked positive and negative words and selected from personality trait descriptors [17]. Positive and negative words were taken from words with above and below average likeableness ratings respectively, and positive and negative words sets were balanced in terms meaningfulness, familiarity, and number of syllables and written language frequency [18]. The word pairs and their selection are described in full elsewhere [4], but in brief, as far as possible word pairs contained words that were semantically linked. The following are example word pairs, “generous, selfish” and “polite, rude”.

### Measures

The primary measure was the BFNE scale [16,19] comprising 12 items assessing beliefs about negative evaluation, which are the core feature of social anxiety, rated on 5-point Likert-type scales (1 = not at all characteristic of me; 5 = extremely characteristic of me). Higher scores indicate greater fear of negative evaluation. Fear of negative evaluation is a defining characteristic of social anxiety; however, FNE is not synonymous with social anxiety and tends to include a wider range of individuals. We therefore included the companion Social Interaction Anxiety and Social Phobia Scales (SIAS, SPS) [20], as measures of social anxiety to establish that the BFNE scores provided a good proxy for social anxiety symptoms. FNE has been found to mediate the association between measures of general distress and the SIAS and SPS [21]. Trait and state anxiety were measured using the Spielberger State-Trait Anxiety Inventory state (STAI-S) and trait (STAI-T) sub-scales [22] and Visual Analogue Scales (VAS; 0 = lowest imaginable level of anxiety; 100 = highest imaginable level of anxiety). To provide psychiatric diagnoses in line with ICD-10 diagnostic categories [23,24], we also included questions pertaining to anxiety, panic and phobia extracted from the revised Clinical Interview Schedule, (CIS-R) [25]. Depression was assessed using the Patient Health Questionnaire (PHQ-9) [26], verbal IQ using the National Adult Reading Test (NART) [27].

### Procedure

After providing informed consent, participants were tested individually in the human testing laboratories in the School of Social and Community Medicine, University of Bristol. A demographics questionnaire, the NART, and a VAS were completed prior to the social evaluation learning task, which was programmed using E-Prime version 2.0 software (Psychology Software Tools Inc., Pittsburgh PA, USA) and completed on a Toshiba Satellite Pro A300-1E7 laptop, Intel Pentium Core2Duo processor, with a 15.4 inch screen. Anxiety (BFNE, SIAS, SPS, STAI-S, STAI-T, and VAS) and depression (PHQ-9) measures were completed after the task. Participants were debriefed, given a chance to ask questions, and reimbursed £10 for their time and travel.

### Social evaluation learning task

The social evaluation learning task was based on probabilistic stimulus-reward learning tasks [4,28], and adapted to incorporate pseudo social interactions. Before starting, the participants were instructed by the experimenter that they would meet a series of six computer personas, during six test blocks. Each persona required the participant to learn one of three social rules (person is liked by persona, neutral, disliked by persona) in one of two conditions: self-referential (e.g., “persona likes me [participant]”), and other-referential (e.g., “persona likes George”). Each persona block consisted of a learning phase, intended to simulate a social interaction, and a global rating phase, intended to measure overall learning, providing a positive response rate and global rating for analysis. In each block, the participants met a new computer persona who presented them with a series of 32 positive/negative word pairs. For each block, the word pairs were selected at random (without replacement) from the list of 64 word pairs. After introducing themselves, the personas instructed the participant to select the word in each pair that corresponded most with what they, the persona, thought about them, the participant (self-referential), or a third person “George” (other-referential). In response to feedback as to whether their choice was correct, participants were instructed to use trial and error to learn whether the personas liked or disliked them / “George”. The 32 word pair trials for the six personas comprised the learning phases.

**Fig 1:**
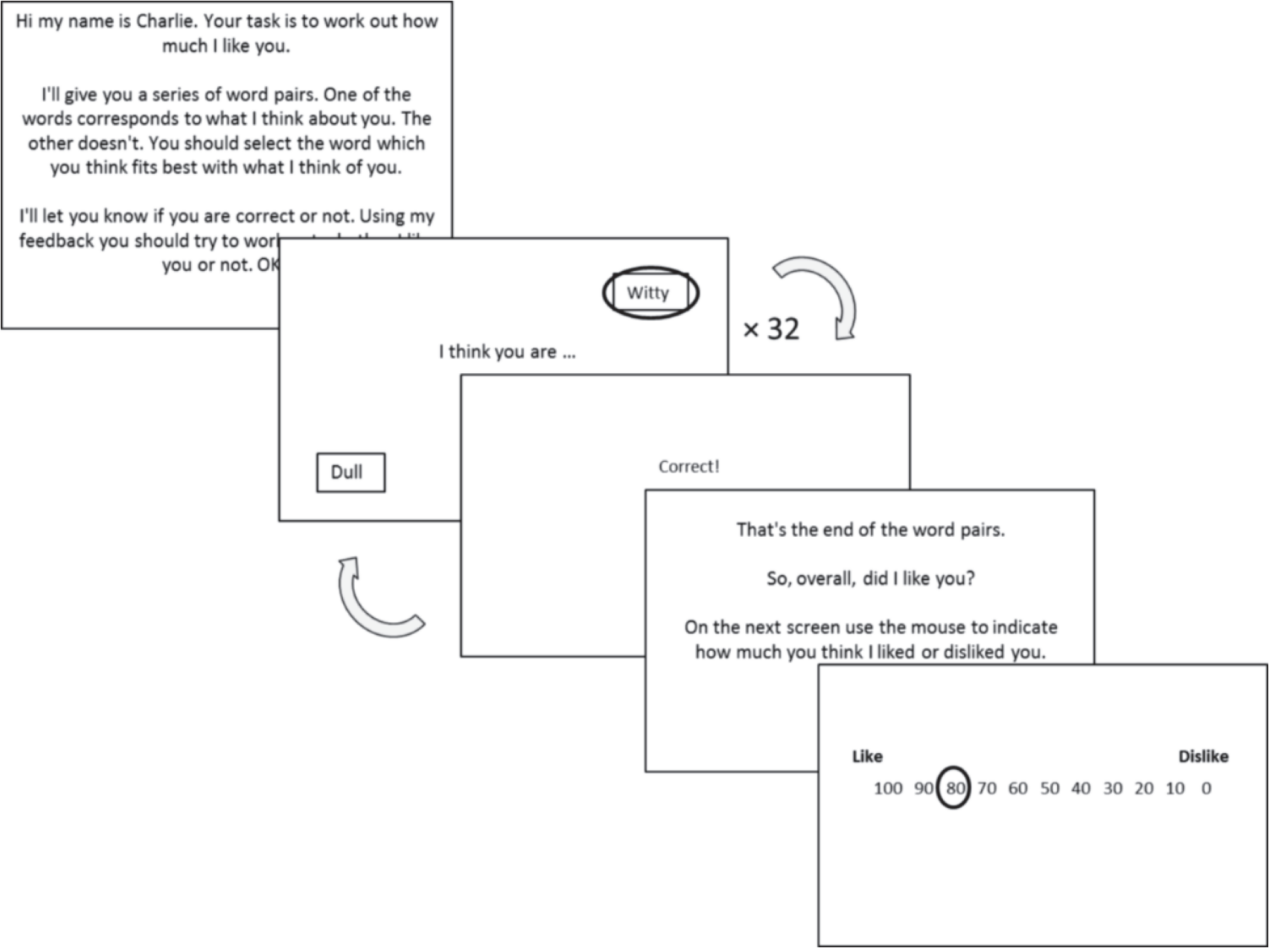
Example of a self-referential rule block in the social evaluation learning task. Each block contains a probabilistic learning phase where 32 word pairs and feedback are presented, and a global rating phase. There were 6 blocks in total, self-liked, self-neutral, self-disliked, other-liked, other-neutral, other-disliked. P[positive word correct] = 0.8, 0.5 and 0.2, for the liked, neutral and disliked rules respectively.

The feedback contingency for the three personas in each condition corresponded to three different rules: like (positive word correct 80%), neutral (positive word correct 50%) and dislike (positive word correct 20%). For the like rules the contingencies were implemented by setting positive words as correct (and negative words as incorrect), and then assigning two in every 10 trials as “false feedback events” at random where the feedback is switched (this was achieved by using a counter which re-set to zero after 10 trials). For example, a positive word choice would be falsely reinforced as “incorrect” and a negative word as “correct”. For neutral, the same applied except for five out of every 10 trials the feedback is switched. For dislike, the negative words were set as correct (and positive words as incorrect), with feedback falsely switched at random for two in every 10 trials. At the end of each block the persona asked the participant to provide a global rating of how much they thought the persona liked them (self-referential) or “George” (other-referential) using a rating scale (0 = completely dislike through to 100 = completely like). These global ratings required the participants to reflect on their previous learning. An example of a test block indicating the nature of the pseudo-social interactions is shown in Fig. 1.

### Data Analysis

The study hypothesis was examined using random effects linear regression modelling (RRM) in the statistical software package Stata 11 (StataCorp, 2009). RRM accounts for the correlation of residuals within individuals due to repeated measures. The outcome for the learning phase was the percentage of positive responses, (n positive words/32) × 100. The outcome for global ratings was percent ‘like’, 0 = completely dislike, 100 = completely like. The regression coefficients (β) for the analyses of both the learning phase and global rating (analysed in separate models) represent the change in percent of positive responses for each unit increase in the predictor variable.

Continuous BFNE score was used as the FNE predictor variable The main effects of FNE, rule (like, neutral, dislike) and referential condition (self, other) on outcome were examined in separate models for learning (percent positive responses) and global ratings. Rule was treated as a categorical variable with three levels (like, neutral, dislike) with ‘like’ as the reference rule and Wald tests were used to test whether rule overall contributed significantly to the models. To test our hypothesis that biased processing in FNE is specific to the self, and to examine how this might vary by rule, we modelled percent positive responses (the learning outcome) as a function of the three predictor variables (FNE, rule, referential condition) and their interaction terms (FNE × referential condition, FNE × rule, and the FNE × referential condition × rule). We then examined global ratings (outcome) as a function of FNE, rule, referential condition, and their interaction terms FNE × referential condition, FNE × rule, and the FNE × referential condition × rule. Finally, we conducted a simple linear regression to examine how well positive response rate predicted global ratings.

**Fig 2:**
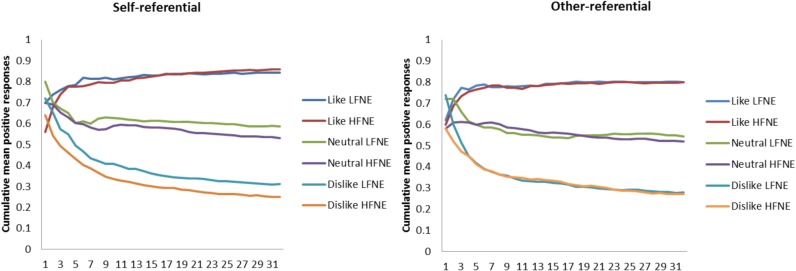
Cumulative mean positive responses over the 32 trials during the learning phase. Learning curves for high (n = 50) and low FNE (n = 50) individuals based on median split of test-day BFNE scores. The high and low FNE groups vary most over the initial trials where high FNE made fewer positive responses. After the initial trials the high and low groups behave similarly except in the neutral and dislike rules in the self-referential condition where the learning curves are clearly separated. The clear differentiation of the curves by rule indicates that individuals were adjusting their response in response to feedback.

To visualise the learning process we plotted the cumulative mean positive responses for the 32 trials for high and low FNE (median split of test-day BFNE scores, Fig. 2). To explore the nature of any FNE-related differences in social-evaluative learning we examined how individuals adjusted their responses following feedback during the learning phases. We looked at correct-repeat behaviour for positive and negative words, that is how often individuals chose the positive word, after being told their previous positive word choice was “correct”. We also looked at incorrect-shift behaviour; that is, how often participants changed the valence of their response after being told they were “incorrect”. To formally test whether FNE was related to differences in these learning outcomes, and whether this varied according to referential condition, we modelled each of the learning outcomes (correct-repeat, incorrect-switch) as a function of FNE, referential condition, rule and the FNE by condition interaction separately for positive and negative words using mixed-effects Poisson regression.

In a simple between-groups design a sample of 100 would have 80% power to detect an effect size *d* = 0.57 at an alpha level of 5%. The primary analysis in this study used BFNE scores as a continuous variable, which increases power. However, testing the specified hypothesis requires testing for interaction, which (depending on the size of the predicted interaction relative to main effect) may decrease power [29]. Our primary hypothesis predicts that the differences in social evalutation learning associated with BFNE will be specific to the self (BFNE × Condition).

## Results

### Characteristics of Participants

Descriptive data are presented in Tables 1 and 2. To illustrate the variability in data across the sample the data are grouped according to BFNE screening groups. However, it should be noted that all analyses treat FNE as continuous to maximise statistical power. The BFNE was administered at screening and test day, and showed good test re-test reliability, IIC 0.94 (95% CI 0.91, 0.96). The test day BFNE scores were used in the analyses.

**Table 1:** Participant characteristics.

	Low (*n* = 33)		Mid (*n =* 32)		High (*n* = 35)	
	M	*SD*	M	*SD*	M	*SD*
Age	28	9	26	6	26	7
BFNE Screening	25	4	40	4	51	4
BFNE Test Day	25	6	43	5	50	4
STAI-state	29	8	40	10	44	9
STAI-trait	32	6	48	10	52	9
SPS	7	6	19	10	24	11
SIAS	11	7	27	10	33	14
PHQ-9	2	3	6	4	7	6
State Anxiety^1^ (pre-task)	13	14	30	19	39	21
State Anxiety^1^ (post-task)	15	12	23	15	39	20
NART	33	6	33	6	33	6
CIS-R Social Phobia: n (%)	0	(0%)	1	(3%)	6	(17%)
Female: n (%)	19	(58%)	21	(66%)	23	(66%)
Student: n (%)	19	(58%)	16	(50%)	19	(54%)
Ever prescribed antidepressants: n (%)	1	(3%)	3	(9%)	3	(9%)
Ever received cognitive therapy: n (%)	2	(6%)	8	(25%)	14	(40%)

Participants grouped by social anxiety based on quartile split of screening BFNE scores. BFNE = Brief Fear of Negative Evaluation scale. STAI = State Trait Anxiety Inventory. SPS = Social Phobia Scale. SIAS = Social Interaction Anxiety Scale. PHQ-9 = Patient Health Questionnaire. NART = National Adult Reading Test. CIS-R = Clinical Interviews Schedule- Revised. AD = antidepressants.

^1^Assesed using a 0–100 visual analogue scale, with increasing score signalling increased state anxiety

**Table 2:** Mean positive response rate and global rating scores for referential condition and rule by screening group.

Task	Condition	Rule	Low (*n* = 33)		Mid (*n =* 32)		High (*n* = 35)	
			M	*SD*	M	*SD*	M	*SD*
Positive response rate^a^	Self	Like (80%)	82	15	89	10	85	10
		Neutral (50%)	60	21	54	17	54	19
		Dislike (20%)	33	22	27	21	24	26
		**Average (50%)**	**58**	**14**	**56**	**11**	**54**	**14**
	Other	Like (80%)	78	14	81	16	82	17
		Neutral (50%)	58	15	51	14	51	19
		Dislike (20%)	30	17	23	15	28	17
		**Average (50%)**	**55**	**10**	**52**	**7**	**54**	**12**
Global rating^b^	Self	Like (80%)	73	21	75	14	70	16
		Neutral (50%)	48	18	42	14	35	17
		Dislike (20%)	29	23	18	16	17	18
		**Average (50%)**	**50**	**13**	**45**	**10**	**41**	**13**
	Other	Like (80%)	72	18	75	18	72	17
		Neutral (50%)	43	14	40	16	40	15
		Dislike (20%)	25	21	24	15	22	16
		**Average (50%)**	**47**	**12**	**46**	**11**	**45**	**10**

^a^ (*n* positive responses/32)*100.

^b^ 0 = complete Dislike, 100 = complete Like. Condition: Self = “persona [likes / neutral / dislikes] me”, Other = “persona [likes / neutral / dislikes] George”. Rules: Like = 80% positive words correct, Neutral = 50% positive words correct, Dislike = 20% positive words correct.

The mean age of participants was 27 years (range 18–49 years). As expected, anxiety and depression increased across the low, medium, and high socially anxious screening groups, as did the number of people who had previously received psychotherapy. Six individuals (17%) in the high group, and one (3%) in the mid group, met the ICD-10 criteria for a diagnosis of social phobia, and 35 individuals across the sample met previously defined clinical thresholds [30]. Participants across the three screening groups were similar concerning verbal IQ and in all other respects.

### Descriptive data

Table 2 shows the unadjusted mean positive response percentage and global rating scores for each rule by referential condition by screening group. Fig. 2 shows the cumulative mean positive responses over the 32 learning trials for each of the rules and referential-condition for high and low FNE. These curves clearly show that participants (on average) adjusted their behaviour on a trial-by-trial basis to learn the rules. The high and low FNE groups seem to vary most over the initial trials where the high FNE group made fewer positive responses. After the initial trials the high and low groups behave similarly except in the neutral and dislike rules in the self-referential condition where the learning curves are clearly separated by high FNE individuals maintaining lower cumulative mean positive responses across the 32 trials.

### Regression

Linear regression modelling the main effects of BFNE, self-referential condition and rule on positive responses found evidence of a main effect of BFNE (β = −0.17, 95% CI -0.32, -0.02, *p* = 0.03), referential condition (β = −2.7, 95% CI -5.3, -0.1, *p* = 0.04), and rule (Wald χ^2^ [2] = 1142, *p* <0.001). Each 10-point increase on the BFNE scale corresponded with a 1.7% (95% CI 0.2%, 3.2%) decrease in positive responses, and participants endorsed 2.7% (95% CI 0.1%, 5.3%) more positive words when learning about themselves relative to others. Participants showed strong discrimination between the rules suggesting that learning had occurred, with the proportion of positive responses generally higher than the actual rule contingency for each of the rules (Table 2).

**Fig 3:**
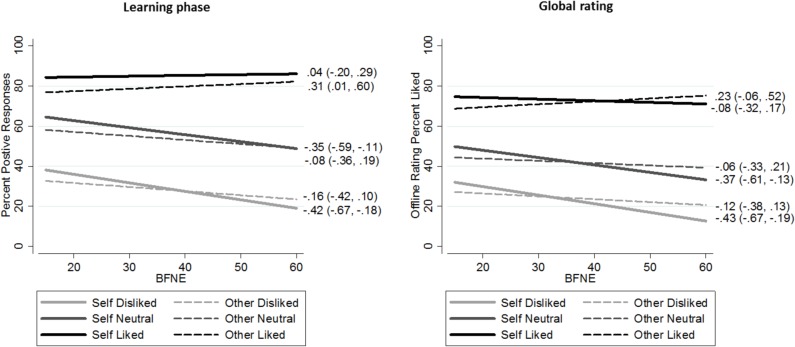
Predicted values for learning phase and global interpretations from regression models testing for differential effects of condition and rule on FNE. BFNE = Brief Fear of Negative Evaluation Scale. Rule contingencies like 80%, neutral 50%, dislike 20%. All regression models were a good fit for the data, each explaining around 64% of overall variance for the learning phase, and around 60% for the global ratings. We predicted the linear relationship between each rule and condition with social anxiety (coefficient and 95% confidence intervals) using the lincom command in Stata. These indicate that learning in the self-referential condition and in the neutral and disliked rules is most associated with FNE, and this holds for both the learning and global rating phases.

Introducing the interactions of interest into the regression model for FNE found evidence for the predicted BFNE × referential condition interaction (interaction term = 0.28, 95% CI 0.01, 0.54, *p* = 0.04); consistent with our hypothesis, BFNE was associated with social learning specific to the self. At low FNE individuals made 9% more positive responses in the self-condition relative to the other-condition. According to the interaction term, each 10-point increase in BFNE score corresponded to a 3 percentage point reduction in positive responses in the self-condition. This is illustrated in Fig. 3; individuals with low FNE made more positive responses in the self-referential condition relative to the other-referential condition, and positive response rate decreased as a function of increasing FNE in the self-referential condition, while it remained more constant in the other-referential condition. This essentially resulted in a reduction in self-favouring with increasing FNE. There was also evidence of a BFNE × rule interaction (*p* = 0.002); increasing FNE was associated with a decreasing proportion of positive responses mostly for the neutral and disliked rules (Table 3; Fig. 3). There was some evidence that this decreasing positive response rate in the neutral and negative rules were strongest for the self-referential condition; Fig. 3 shows the predicted linear relationship between each of the six rule-conditions with FNE calculated using the lincom command in Stata. These linear terms indicate that responses in the self-dislike rule (i.e., reference categories) varied most with FNE (β = −0.42, 95% CI -0.67, -0.1.8), slightly less in the self-neutral rule (β = −0.35, 95% CI -0.59, -0.11), and did not vary with FNE in the self-positive rule (β = 0.04, 95% CI -0.20, -0.29). However, the three-way BFNE × referential condition × rule interaction term did not meet the criterion for statistical significance (*p* = 0.07; Table 3). The predicted values in Fig. 3 also suggest individuals with extreme low FNE were poor at learning the neutral and disliked rules in the self-condition; their proportion of positive responses was about 10% greater relative to the other-condition and about 20% greater than the actual rule contingency. As FNE increased this positive self-referential bias was attenuated. The predicted positive responses for those with highest FNE were similar to the actual rule contingencies suggesting that these individuals were accurate in learning across all rules. There was no evidence that the interaction of interest (i.e., FNE × condition) was modulated by age or gender (FNE × condition × age = 0.00, 95% CI -0.01, 0.00, *p* = 0.33; FNE × condition × gender = −0.04, 95% CI -0.15, 0.07, *p* = 0.51). Controlling for trait depression did not affect the results (coefficients remain the same as in Table 3); controlling for anxiety slightly increased the effect of FNE in the reference self-dislike condition (β = −0.5, 95% CI -0.8, -0.2, *p* = 0.003), but had no effect on the interaction terms given in Table 3. Consistent with our hypothesis, we found similar results when we replaced BFNE for other social anxiety variables, the SIAS and SPS (SIAS × condition β = 0.30, 95% CI 0.04, 0.56; *p* = 0.02, SPS × condition β = 0.41, 95% CI 0.07, 0.75, *p* = 0.02).

**Table 3:** Regression coefficients (95% CI) from regression models testing for interactions of rule and self-referential condition with BFNE for the positive response proportion and global ratings in the social learning task.

	Response Rate			Global Rating		
Variable	*β*	95% CI	*p*	*β*	95% CI	*p*
BFNE	−0.4	−0.7–0.2	0.001	−0.4	−0.7–0.2	0.001
Condition	−9	−18 0.4	0.06	−9	−18–0.3	0.04
Rule			<0.001			<0.001
*Neutral*	25	14 36		17	6 28	
*Like*	39	28 50		37	26 48	
BFNE × Condition	0.3	0.01 0.5	0.04	0.3	0.06 0.6	0.02
BFNE × Rule			0.002			0.03
*BFNE × Neutral*	0. 07	−0.2 0.3		0.06	−0.2 0.3	
*BFNE × Like*	0. 5	0.2 0.7		0.4	0.1 0.6	
BFNE × Rule × Condition	−0.002	−0.005 2 ×10^−4^	0.07	−0.001	−0.004 0.001	0.4

BFNE = Brief Fear of Negative Evaluation scale. Dislike is the reference rule, Self is the reference condition, therefore for every 1-point increase in BFNE positive responses decrease by 0.4 in the self-dislike rule. *p-*values for variables with 3 levels based on Wald tests. Values < 1 reported to 1 significant figure.

**Fig 4:**
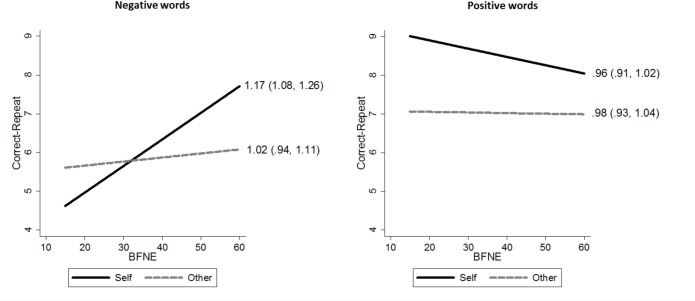
Correct-repeat behaviour during the learning phase. Predicted values from the Poisson regression models testing for the referential-condition by FNE interaction on correct-repeat behaviour during the learning phase in separate models for negative (left) and positive (right) words. We used the lincom command in Stata to estimate the relationship (rate ratio and 95% confidence intervals) between correct-repeat behaviour and a one standard deviation increase in BFNE (1 s.d. corresponds to 11.5 BFNE points) by referential condition. FNE was selectively associated with self-referential negative-correct-repeat responses, with the coefficient indicating a 17% increase for each 11.5 point increase in BFNE, *p* < 0.001. There was no evidence to suggest that negative-correct-repeat behaviour in the other other-referential condition, or that positive-correct-repeat behaviour in either condition, varied with FNE, *p*’s > 0.2.

Mixed effects Poisson regression indicated that FNE was associated with an increase in correct-repeat responses for negative words (rate ratio = 1.17, 95% CI 1.08, 1.26, *p* < 0.001), which corresponds to a 17% increase in negative-correct-repeat behaviour for each standard deviation increase in BFNE score (one standard deviation corresponds to 11.5 BFNE points). There was also evidence for the interaction with referential condition (FNE × condition = 0.87, 95% CI 0.82, 0.93, *p* < 0.001) indicating that the increase in correct-repeat behaviour was specific to the self-referential condition and not present in the other-referential condition (Fig. 4). There was no evidence to suggest that positive-correct-repeat behaviour varied with FNE (rate ratio = 0.96, 95% CI 0.91, 1.02, *p* = 0.20; FNE × condition = 1.02, 95% CI 0.96, 1.08, *p* = 0. 58). Incorrect-switch behaviour was not related to FNE for positive words (rate ratio = 0.97, 95% CI 0.95, 1.12, *p* = 0.41; FNE × condition = 0.97, 95% CI 0.89, 1.07, *p* = 0.57) or negative words (rate ratio = 0.97, 95% CI 0.90, 1.04, *p* = 0.40; FNE × condition = 1.04, 95% CI 0.95, 1.14, *p* = 0.36).

### Global Rating Phase

There was evidence of main effects of BFNE (β = −0.16, 95% CI -0.32, -0.01, *p* = 0.03), and rule (Wald χ^2^ [2] = 999, *p* <0.001), but not self-referential condition (*p* = 0.5), on global ratings. Similar to the learning responses rate each 10-point increase on the BFNE scale corresponded with a 1.6% (95% CI 0.1%, 3.2%) decrease in like ratings. Participants’ global ratings showed clear discrimination between the rules, further confirming that learning had occurred during the learning phase (see Table 2 for means). Testing for interactions found evidence of the hypothesised BFNE × referential condition interaction (interaction term = 0.31, 95% CI 0.06, 0.56, *p* = 0.02), and evidence of BFNE× rule interaction (Wald χ^2^ [2] = 7, *p* = 0.03), but no evidence for the 3-way interaction (*p =* 0.4). Consistent with our hypothesis, increased FNE was associated with decreased like ratings most strongly in the self-condition (Table 3; Fig. 3). Like ratings also decreased most strongly in the neutral and negative rules, as indexed by the linear terms presented in Fig. 3.

Responses during the learning phase were positively associated with the global ratings in a simple regression, β = 0.74, 95% CI 0.69, 0.79, *p <* 0.001.

## Discussion

As hypothesised, the results suggest that FNE was associated with differences in learning of social evaluation specifically about the self. On average, as FNE increased participants made fewer positive (and thus more negative) responses in the self-condition. Less expected was the (albeit weak) evidence that this self-referential effect was strongest for the neutral and disliked rules. At low FNE, learning neutral and negative self-evaluation was particularly poor, and accuracy in these rules increased with FNE. By contrast, learning positive evaluation was uniformly good at around 80% across all levels of FNE. Examining feedback learning behaviours indicated that these FNE-related differences in social-evaluative learning arise primarily due to differences in correct-repeat responses to negative words, suggesting that high FNE individuals are more sensitive to feedback confirming negative word choices. A similar pattern of results was observed for the global ratings, confirming that the positive contingencies were successfully learned by participants.

### Self-referential bias and self-favouring

Positive self-referential biases are thought to be protective for mental health (Taylor & Brown, 1994). In our sample, individuals from the lower half of the FNE distribution showed a positive self-referential bias in two ways: first, their positive response rate exceeded that of the actual self-neutral and self-dislike rule contingencies by some margin, suggesting they were overly optimistic given the actual evidence, and; second, their positive response rate was substantially higher in the self-condition relative to the other-condition. The selectivity of this optimistic learning indicates that social evaluation learning *per se* is not biased by FNE, but that FNE acts selectively on self-referential learning. Individuals with relatively low FNE seemed to disregard feedback suggesting negative evaluation about the self. This would presumably increase confidence during social interactions, produce better social outcomes, and reduce the availability of negative information for later rumination. This may be one way a positive self-referential bias may protect mental health. By contrast, individuals with high FNE learnt positive and negative information more equally, indicating more accurate, but perhaps less healthy, social evaluation learning. This has parallels with the loss of positive bias has been described in the “depressive realism” literature [31,32].

Responses of low FNE individuals indicated that they inferred markedly more favourable opinions of their own social evaluation relative to the other. By contrast at high FNE this self-favouring effect was abolished. Some theorists suggest that social anxiety behaviours may have evolved as adaptive processes to maintain social hierarchy [33], and a loss of this self-favouring may contribute to a sense of social inferiority.

### Positive, neutral, and negative evaluation

We have previously reported that low social anxiety individuals made substantially fewer errors learning positive than negative evaluation, and that this positive bias diminished as social anxiety increased [4]. Of note however, was that this effect seemed driven by similar changes in both rules; decreased errors in the negative rule, which equated to fewer positive responses, and increased errors in the positive rule which also equated to fewer positive responses (due to the binary positive or negative word choice). In the present study, however, learning positive evaluation was uniformly good and unrelated to FNE (Fig. 3). By contrast, we found that FNE was most associated with learning neutral and negative self-evaluation (Figs. 2 and 3). As this finding was not anticipated it should be considered as hypothesis-generating and thus requires further replication before strong conclusions can be drawn. However, examining learning behaviour indicated that these selective rule effects were likely driven by differences in correct-repeat behaviour for self-negative (but not positive) words; at low FNE individual made very few (~5) negative-correct-repeat behaviours, compared to their positive-correct-repeat behaviours (~9), whereas at high FNE correct-repeat behaviours increased considerably for negative words, whilst remaining relatively constant for positive words, resulting in similar numbers for both negative and positive words (~8; Fig. 4). This effect was specific to self-referential words; negative-correct updating in the other-referential condition did not vary with FNE (Fig. 4). This suggests that FNE might be associated with differences in the weighting given to correct feedback to negative self-referential words, with low FNE individual discounting or disregarding such feedback so that they fail to update their belief about how much they are disliked. By contrast high FNE individuals might weight such feedback similar to positive-correct feedback and thus update their beliefs of how much they are liked and disliked more equally. This might also explain why positive evaluation was unrelated to FNE; there are few opportunities for negative-correct-repeat behaviours. It also explains the dose-response relationship with neutral and negative rules where the association with FNE increased as the probability of negative words being correct increased. Further work is needed to examine these new hypotheses.

### Global Reflections

Responses during the learning phase predicted global ratings, indicating that learning had occurred. However, global ratings were lower than the respective positive response rates (Fig. 3). This may reflect the influence of reflective appraisal in global interpretations, and lends support to dual-process models [10]. Our findings may also help to integrate the seeming discrepancy in the literature between “absence of positive” biases observed in learning paradigms [4–9] and “negative biases” observed in more global interpretive paradigms [14,15]. Negative interpretations of social interactions are thought to increase social avoidance [34], and contribute to cycles of negative rumination [14]. Consistent with previous research [35], we found that global ratings were more negative (i.e., person rated as less liked) as FNE increased. At high levels of FNE, ratings were lower than the true rule contingency, indicating unduly gloomy interpretations given the actual evidence, and this effect was strongest in the self-referential condition. For high FNE, ratings in the self-condition were lower compared to the other-condition (Fig. 2). Socially anxious individuals report feelings of social inferiority and low social status [36]. The biases in social evaluation learning we describe could logically serve to maintain such feelings.

### Clinical Implications

Our results provide support for the aspects of current cognitive therapies and cognitive models which emphasise the role of FNE and self-schemata in maintaining symptoms [15]. Challenging an individual’s cognitions by switching the self-referential perspective to that of a third person is a widely used technique in cognitive behavioural therapy and our results suggest this should be particularly effective in social anxiety where FNE is high. However, in cognitive therapy socially anxious people are helped to more realistically appraise situations, and often to focus on signs of being liked. Our data suggest two things: first, that learning positive evaluation (i.e., person is liked) was uniformly good and least related to social anxiety, and; second that low anxious people are actually positively biased in relation to themselves. If our findings generalise to real social interactions, and clinically ill groups, then they may suggest that encouraging socially anxious people to disengage from negative information and cultivate a positive interpretation bias may be therapeutic. Evidence for modifying positive interpretation biases is preliminary [37] but our results suggest this as a possible avenue for treatment.

Research has indicated that people with social anxiety may feel threatened by positive social feedback, possibly because it may mean that others will have higher expectations of them in the future [38]. Furthermore, modern cognitive theories suggest that fear of positive evaluation is also a core feature of social anxiety [39]. Our findings offer little support to this, as social anxiety was least associated with differences when learning the liked rule. The differences were strongest in the ambiguous neutral and disliked rules, where high social anxiety was associated with greater sensitivity to learning negative evaluation indexed by increased negative-correct-repeat behaviour.

### Strengths and limitations

The main strength of this study is the innovative experimental design. Using this approach, our study is the first to establish that the social evaluation learning biases associated with social anxiety are more strongly related to self-referential information. The design is also a limitation; the task was analogue and we cannot be sure that we are truly tapping a real social processing effect, or whether the results may reflect a reporting bias. For example, individuals with high FNE may feel embarrassed about selecting positive words, especially about themselves. However, we can rule this form of bias out as all participants learnt the positive rule and this was least related to FNE. Similarly, the extent to which participants felt socially engaged during the task is unclear, and further research is needed to assess whether performance on this analogue task taps processing which occurs in real social interactions. However, participants often respond to interactions with computer personas in a similar manner to ‘real’ people, for example with comparable negative feeling following ostracism [40].

A further limitation is the analogue sample. We cannot be sure how applicable the results are to social anxiety and the clinical disorder. Nonetheless, while we focused on FNE, many of our participants were highly symptomatic; seven met the ICD-10 criteria for social phobia diagnosis and 35 met previously defined clinical thresholds on social anxiety scales [30]. The design we adopted also reduces the possibility of introducing a selection bias that could happen with case-control studies in which clinical cases are compared with people without the condition [41]. Further, modelling FNE as a continuous trait suggests a linear relationship between FNE and social evaluation learning. This is consistent with the idea that social anxiety fears and social anxiety disorder lie upon a continuum of severity [42].

## Conclusions

As FNE increased, learning negative social evaluation improved, that was specific to learning about the self, and related to increased sensitivity to feedback indicating that negative self-referential endorsements were correct. These findings support cognitive models of social anxiety that suggest activation of self-schema may bias social processing in favour of less positive conclusions [15]. During the learning phase high FNE individuals were more accurate than the ‘positively’ biased low FNE individuals. This raises the prospect that the absence of a ‘positive’ self-referential bias (potentially maintained by selective discounting of negative self-referential feedback) may be a factor that increases the risk of social anxiety. If, as our evidence suggests, social evaluation learning processes are biased by FNE and this contributes to social anxiety, then treatment plans which address such learning may be clinically useful.
